# Effects of Growth Medium and Inoculum Size on Pharmacodynamics Activity of Marbofloxacin against *Staphylococcus aureus* Isolated from Caprine Clinical Mastitis

**DOI:** 10.3390/antibiotics10111290

**Published:** 2021-10-22

**Authors:** Augusto Matías Lorenzutti, Manuel Ignacio San Andrés-Larrea, Emilio Fernández-Varón, María del Pilar Zarazaga, Ana María Molina-López, Juan Manuel Serrano-Rodríguez

**Affiliations:** 1 Facultad de Ciencias Agropecuarias, IRNASUS CONICET—Universidad Católica de Córdoba, Córdoba X5016DHK, Argentina; matiaslorenzutti@ucc.edu.ar (A.M.L.); 0205517@ucc.edu.ar (M.d.P.Z.); 2 Department of Pharmacology and Toxicology, Faculty of Veterinary Medicine, Universidad Complutense de Madrid, 28040 Madrid, Spain; misanand@vet.ucm.es; 3 Center for Biomedical Research (CIBM), Department of Pharmacology, University of Granada, 18071 Granada, Spain; emiliofv@ugr.es; 4 Instituto de Investigación Biosanitaria de Granada (ibs.GRANADA), 18071 Granada, Spain; 5 Department of Anatomy, Comparative Pathology and Toxicology, University of Córdoba, 14071 Córdoba, Spain; ft2moloa@uco.es; 6 Department of Nursing, Pharmacology and Physiotherapy, Pharmacology Area, Faculty of Veterinary Medicine, Universidad de Córdoba, 14071 Córdoba, Spain

**Keywords:** marbofloxacin, *S. aureus*, inoculum size, lactating goats, clinical mastitis, non-linear mixed effect modeling, pharmacodynamics

## Abstract

*Staphylococcus aureus* (*S. aureus*) is an important pathogen that causes clinical mastitis in goats and produces infections difficult to cure. Different antimicrobials as fluoroquinolones have been used against *S. aureus*. However, the studies developed to evaluate the bacterial drug interaction only have used the MIC as a single reference point with artificial growth media. The aims of this study were to describe the effect of marbofloxacin on *S. aureus* isolated from mastitis goats’ milk by different approaches as the minimum inhibitory and bactericidal concentrations (MIC and MBC) in cation adjusted Mueller–Hinton broth (CAMHB), serum and milk of goats at two inoculum sizes of 10^5^ and 10^8^ CFU/mL, the determination and analysis of the time kill curves (TKC) by non-linear mixed effect models in each growth medium and inoculum size, as well as the estimation of their pharmacokinetics/pharmacodynamics (PK/PD) cutoff values. The results obtained indicate that MIC values were higher and increases 2,4-fold in serum and 3,6-fold in milk at high inoculum, as well as the EC_50_ values determined by each pharmacodynamics model. Finally, the PK/PD cutoff values defined as fAUC_24_/MIC ratios to achieve clinical efficacy were highly dependent on inoculum and growth medium, with median values of 60–180, especially at high inoculum in milk, suggesting that further studies are necessary to evaluate and optimize the best therapeutic strategies for treating *S. aureus* in lactating goats.

## 1. Introduction

*Staphylococcus aureus* is an important pathogen in ruminant species that can colonize the udder from where it can infect the mammary gland epithelium causing mastitis [1]. In small ruminants, it is responsible for approximately 80% of cases of clinical mastitis in dairy ewes and is the most common cause in dairy goats [2,3]. Moreover, this disease is a highly relevant issue due to the economic losses for producers, particularly with respect to bacteriological quality of milk [4].

*S. aureus* intramammary infections (IMI) are difficult to cure because bacteria remain in milk but penetrate udder tissue and cause deep infection. For this purpose, drug administration requires accumulation and maintenance of effective concentrations in milk, blood and udder tissue [5]. The extent to which a drug has access into these compartments depends on its pharmacokinetic properties: lipophilicity, ionization, and binding protein [6]. In this regard, fluoroquinolones exhibit good properties as high bioavailability and lipophilicity, large volume of distribution, low protein binding and milk excretion by passive diffusion, and active transport by BCRP protein [7]. Therefore, these drugs distribute well into mammary glands with milk concentrations similar or higher than serum concentrations [8].

Marbofloxacin is a fluoroquinolone developed exclusively for veterinary use, which presents high activity against gram negative and positive pathogens and *Mycoplasma* spp. From a pharmacodynamic point of view, it is a bactericidal concentration-dependent agent and acts by inhibiting bacterial DNA topoisomerases II and IV [9]. As with other fluoroquinolones, it also has a prolonged post-antibiotic effect (PAE) [10].

This agent is approved in the European Union (EU) for use in lactating dairy cattle and pigs for treatment of mastitis and respiratory disease, with well-established maximum residue limits [11]. However, these uses may be extended to other minority species, such sheep or goats through an extra-label use, where the studies are limited. Moreover, it is included in group B of the antibiotics categorization of the EU, which includes agents that should only be used based on the results of antibacterial susceptibility testing for the treatment of clinical conditions with no alternative in other categories [12].

Antimicrobial dosage regimens are mostly based to achieve serum or tissue concentrations above the minimum inhibitory concentration (MIC) for the target pathogen [9]. However, MIC has known disadvantages. First, artificial media composition differs significantly from body fluids. Second, protein binding and tissue distribution are overlooked. Third, other factors such as the immunocompetence of the host and the post-antibiotic effect are also ignored. Fourth, the standard inoculum size ≈ 10^5^ CFU/mL ignores the bacterial burden in different clinical infections can achieve a wide range of bacterial densities, e.g., the udder infected can excrete *S. aureus* in milk at levels of 10^4^–10^8^ CFU/mL [4]. For these reasons, different authors suggest other approaches to describe the pharmacodynamics profile of the drug–microorganism interaction such as the use biological fluids, including milk or serum, for susceptibility tests [5,13], time kill curves (TKC) with different inoculum sizes [3,14], and parameters derived from the Hill function obtained with non-linear mixed effect models [14,15].

For antimicrobial drugs, the pharmacokinetic/pharmacodynamics (PK/PD) approach is a useful tool to optimize dose regimens and establish correlations with ratios as a surrogate marker of clinical efficacy. In the case of fluoroquinolones, the PK/PD index selected is the ratio of the area under the plasma time curve (AUC) for free drug to MIC, expressed as *fAUC*/MIC, with f representing the unbound plasma drug concentration [16]. Typical values for *fAUC*/MIC ratios close to 100–125 for gram-negative and 30–55 for gram-positive pathogens have been described as cutoff values [9]. At this point, it is necessary to note that one of the best ways to establish a PK/PD index is by using TKC because their quantitative analysis, after in vitro assays, provides more information regarding drug–bacteria interaction than the use of MIC as a single point of reference [15].

TKC modeling combined with PK/PD models is common in human medicine [17]; however, to our knowledge these models have been minimally applied in veterinary medicine, with some exceptions [15]. The analysis of these curves is complex due to the large number of ordinary differential equations (ODE) and parameters that are generated, but which are necessary for an estimation of the cutoff points. A powerful strategy to analyze TKC is the use of non-linear mixed effects modeling. Their main advantages are the estimation of population parameters, the inter-individual variability (IIV), and the effect of different covariates on the estimation of the parameters. On the other hand, simulations derived from this methodology can include multiple scenarios, such as different bacterial populations, low or high inoculum, or single and multiple dose regimens that could be difficult to conduct in in vivo experimental studies [14,15,17].

Thus, the objectives of the present study were as follows: (1) to obtain the minimum inhibitory concentration and minimum bactericidal concentration, *MIC* and *MBC*, of marbofloxacin against *S. aureus* isolated from mastitis goats’ milk in artificial medium, serum and milk from goats using two inoculum sizes close to 5 × 10^5^ and 5 × 10^8^ CFU/mL; (2) to determine the bactericidal activity using TKC over 24 h across different growth mediums and inoculum sizes by non-linear mixed effect modeling; and (3) to combine the previous models to determine potential PK/PD cutoff points in each growth medium and inoculum size. 

We hypothesized that combining high inoculum size of *S. aureus* in milk compared to serum or artificial medium could lead to greater reduction in activity of marbofloxacin. Results obtained from this study could provide additional understanding of in vitro marbofloxacin pharmacodynamics against *S. aureus* isolated from small ruminants.

## 2. Materials and Methods

### 2.1. Bacterial Isolates, Culture Medium and Antimicrobials

A total of 12 *S. aureus* isolated from the milk of goats with clinical mastitis were used [18]. Bacterial isolates were obtained from different farms in Spain and Portugal in 2012, and were stored at −80 °C in a nutrient broth enriched with 15% glycerol until assayed (see Supplementary Material for more information). 

The growth mediums for the assays were cation adjusted Mueller–Hinton broth (CAMHB, Fluka analytical, Sigma–Aldrich, Madrid, Spain), filtered and sterilized serum obtained from the blood of healthy dairy goats, as well as milk, that was sterilized by autoclaving, at 120 °C for 5 min, in order to avoid milk denaturalization. No bacterial growth was observed after plating the sterilized milk in Mueller–Hinton agar. The milk was buffered with 100 mM HEPES (Sigma–Aldrich, Madrid, Spain). 

Marbofloxacin was obtained from Fluka analytical, Sigma–Aldrich, Madrid, Spain. Stock solutions made and stored at −80 °C until assayed. 

### 2.2. MIC and MBC Measurements 

For each isolated drug, MIC determination was carried out in parallel in each medium using the broth macrodilution method for low and high inoculum according the Clinical and Laboratory Standards Institute protocol (CLSI) [19]. After overnight incubation in CAMHB agar plates at 37 °C, cells were suspended in 50 mL of CAMHB with shaking at 200 rpm for 24 h at 37 °C. Bacterial suspensions were further diluted to the test tubes to achieve a final inoculum close to 10^5^ or 10^8^ CFU/mL [20]. The tubes contained antimicrobial dilutions ranging from 0.03 to 128 μg/mL. The final volume was 1 mL. Assays were incubated at 37 °C with shaking at 200 rpm for 24 h. However, bacterial growth was not visible in milk due to opacity of emulsion; thus, direct plate counting was used. For this purpose, samples were taken from each tube at 0 and 24 h to count viable bacteria, as have been previously described [21]. MIC was defined as the lowest concentration of antibacterial to which the count gave a value less than initial inoculum size after 24 h. The minimum bactericidal concentration (*MBC*) was established by plate count as the concentration of antibacterial to reduce a −3 *log*_10_ (99.9% killing) in the initial inoculum, in accordance with CLSI guidelines [22]. 

### 2.3. Time Kill Curves 

Time kill curves (TKC) were determined following the CLSI guidelines [22]. Two trials were performed at low and high inoculum. 

Low inoculum assay: for each isolate of *S. aureus* overnight cultures were diluted in each growth medium and incubated for 120 min at 37 °C to obtain final inoculum close to 10^5^ CFU/mL. Subsequently, they were transferred to test tubes at 37 ºC with antimicrobial dilutions at 0 (control), 0.125, 0.25, 0.5, 1, 2, 4, 8 and 16 multiples of the MIC value previously obtained, and aliquots of 25 μL were sampled at 0, 1, 2, 4, 8, 12 and 24 h from each test tube. 

High inoculum assay: the same methodology describe at low inoculum was carried out but using a final bacterial density close to 10^8^ CFU/mL. Antimicrobial dilutions at 0 (control), 0.5, 1, 2, 4, 8, 16, 32 and 64 multiples of MIC were used and aliquots of 25 μL were sampled at 0, 1, 2, 4, 8, 12 and 24 h from each test tube. 

In both assays, bacterial counts were determined by serial dilution and culture on CAMHB agar plates at 37 °C. The lower limit of detection was 40 CFU/mL. Moreover, each assay was performed in duplicate on two different days and the averaged value (geometric mean) was calculated from each sampling time. 

In this research, three growth mediums and two inoculum sizes were used and 6 data sets were obtained at low and high inoculum in CAMHB, serum and milk with 108 TKC per data set.

A more detailed description of TKC determination and tabulation of data sets can be found in the Supplementary Material section.

### 2.4. Drug Stability 

To ensure the stability of drugs over the study, another assay was performed in parallel with the TKC. Test tubes with 0.25, 2, 16 and 64× MIC in each medium were evaluated. Samples of 100 μL were taken at 0, 2, 8 and 24 h and were measured by the HPLC method, previously described by our research group [23].

Drug concentrations determined at 2, 8 and 24 h were not different from control groups at 0 h with a variation close to 2–5%. Thus, a reduction of marbofloxacin concentrations by degradation was not detected. Similar results for ciprofloxacin have been obtained, suggesting a high chemical stability in this class of drugs [20]. 

It is known that in vitro pH of milk with mastitis pathogens can decrease between 1–1.5 fold [24,25]. Moreover, the acidity can reduce the antibacterial activity of fluoroquinolones [26]. For this reason, pH in milk was measured previously in another assay. Values close to 5.8 and 5.2 after 24 h for low and high inoculum were obtained. Therefore, milk was buffered with HEPES at 100 mM [26]. 

### 2.5. Pharmacodynamic Modeling

Before the development of the models, drug concentrations in each fluid were transformed to free concentrations using the unbound fractions in plasma and milk, previously determined in our laboratory, with values of 0.71 for milk, and 0.73 for serum [23]. On the other hand, it was also determined in CAMHB with a value close to 0.923. Therefore, it could be assumed that total and unbound concentrations of marbofloxacin in CAMHB were similar, as previously described [13]. Following these indications, TKC used in the present study represents bacterial growth (dependent variable) versus free concentrations of marbofloxacin (regressor). 

For each growth medium, all TKC were simultaneously analyzed using non-linear mixed effect modeling with Monolix 2020R1 suite software (Antony, France: Lixoft SAS, 2020). Each parameter of the final model was described in the general form:

(1) $$\mathrm{Individual}\;\mathrm{parameter}=\theta_{pop}\cdot e^{\eta \theta }\cdot e^{\beta cov\theta }$$

where *θ_pop_* is the parameter estimate, *ηθ* is the inter-individual variability (IIV) and *βcovθ* is the covariate parameter, continuous or categorical, which had an influence on the determination of the parameter and was included in the final model [17]. Covariates were evaluated in order to determine its effects on the estimated parameters and were included in the final model if showed statistical significance (*p* < 0.05) and reduced the variability and the likelihood ratio tests (LRT) as −2·log-likelihood (−2 LL), Akaike information criterion (AIC), and Bayesian information criterion (BIC) [27]. Two covariates were investigated: the *MIC* of each, isolated as a continuous covariate and the size of the initial inoculum (low or high) as a categorical covariate. High inoculum was selected as reference.

To analyze the data, two previously published semi-mechanistic models were used with some modifications [28,29]. 

In a first analysis, a simple one population bacterial model was used to fit the data and expressed with the following equation:

(2) $$\frac{dN}{dt}=k_{g}\cdot \left(1-\frac{N}{N_{MAX}}\;\right)\cdot N-K_{MAX}\cdot \frac{C^{\gamma }}{C^{\gamma }+EC_{50}^{\gamma }}\;\cdot N$$

where $\frac{dN}{dt}$ is the change of the bacterial populations as a function of time, *k_g_* is the growth rate constant followed by a logistic function to limit growth to a maximum total bacterial population size observed *N_MAX_* [17]. Bacterial drug interaction was modeled using a Hill model where *K_MAX_* is the killing rate constant, *EC*_50_ is the free drug concentration to achieve 50% of killing, *γ* is the sigmoidicity constant of the concentration effect curve and *C* is the free drug concentration multiple of *MIC* used is each assay. 

However, a delayed growth was observed in the initial phases [28]. As a consequence, an exponential function was used to describe this delay and was expressed as *dk_g_* and included in the final model:

(3) $$\frac{dN}{dt}=k_{g}\cdot \left(1-e^{-dkg\cdot t}\right)\cdot \left(1-\frac{N}{N_{MAX}}\;\right)\cdot N-K_{MAX}\cdot \frac{C^{\gamma }}{C^{\gamma }+EC_{50}^{\gamma }}\;\cdot N$$

This model is a variation of a classic one compartmental bacterial model [30], which has been widely used by many research groups [17,31].

In a second analysis, a two compartmental bacterial model was used, including two preexisting populations, named susceptible *S* and less susceptible *R* and derived from a bacterial measured as *N* = *S* + *R* [32]. Both subpopulations were not interconverted, and was assumed to have the same killing rate constant *K_MAX_* but different free drug concentration to achieve 50% of killing (*EC*_50*S*_ and *EC*_50*R*_), as well as different grow rate constants (*k_gS_* and *k_gR_*). The final model was expressed as a system of differential equations:

(4) $$\frac{dS}{dt}=k_{gS}\cdot \left(1-e^{-dkg\cdot t}\right)\cdot \left(1-\frac{S+R}{N_{MAX}}\right)\cdot S-K_{MAX}\cdot \frac{C^{\gamma }}{C^{\gamma }+EC_{50S}^{\gamma }}\;\cdot S$$

(5) $$\frac{dR}{dt}=k_{gR}\cdot \left(1-e^{-dkg\cdot t}\right)\cdot \left(1-\frac{S+R}{N_{MAX}}\right)\cdot R-K_{MAX}\cdot \frac{C^{\gamma }}{C^{\gamma }+EC_{50R}^{\gamma }}\;\cdot R$$

**Figure 1:**
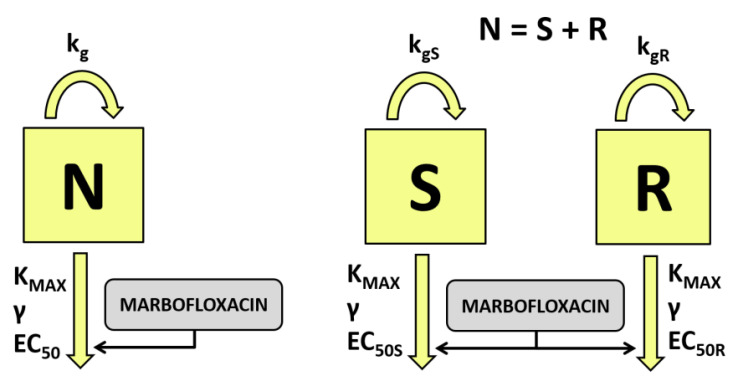
Semi-mechanistic pharmacodynamic models for time kill curves modeling. On the left, a simple one population bacterial model was used to fit the data. Bacteria growing at growth rate constant are exposed to marbofloxacin concentrations with a maximum killing rate constant *K_MAX_* and free drug concentration to achieve 50% of killing *EC*_50_. On the right, two compartmental bacterial model was used. Two populations *S* and *R* of bacteria growing at growth rate constants of *k_gS_* and *k_gR_* are exposed to marbofloxacin with a killing rate constant *K_MAX_* and different drug concentration to achieve 50% of killing (*EC*_50*S*_ and *EC*_50*R*_).

The models used in this study have been previously developed and validated with fluoroquinolones against *S. aureus* and other pathogens; specifically, they have been used in artificial and biological mediums [33,34,35]. Both models are derivations of model M1 and M3 indicated for these studies by other research groups [29]. On the other hand, some modifications were included for a good convergence of the model, *k_gR_* and *EC*_50*R*_ were expressed as proportionality factors to *k_gS_* and *EC*_50*S*_ as *k_gR_* = *f_g_* · *k_gS_* and *EC*_50*R*_ = *f_S_* · *EC*_50*S*_, respectively. Both models are presented in Figure 1. 

### 2.6. Simulations and PK/PD Relationships

After modeling the TKC with both population models, the results obtained were imported to Simulx, a simulation package included into the Monolix 2020R1 suite software. For each population model and growth medium, 1000 TKC were simulated, including *MIC* and inoculum size as covariates. Next, the PK-PD cutoffs to marbofloxacin were evaluated. 

Firstly, from each TKC simulated, the *log*_10_ difference between the bacterial count after 24 h of exposition and the initial bacterial count (bacterial population time zero) was calculated and defined as drug effect [13]:

(6) $$E=Log{\left(N\right)}_{24h}-Log{\left(N\right)}_{0h}\;$$

Secondly, the *fAUC*_24_/*MIC* ratio was obtained by multiplying each free concentration by 24 h and dividing the *fAUC*_24_ by the *MIC* as has been described [15]. 

Finally, the relationship between the *fAUC*_24_/*MIC* ratios calculated and the drug effect was determined using the inhibitory *I_MAX_* model:

(7) $$E=E_{0}-I_{MAX}\cdot \frac{{\left(\frac{AUC_{24}}{MIC}\right)}^{\gamma }}{{\left(\frac{AUC_{24}}{MIC}\right)}^{\gamma }+{\left(\frac{AUC_{24}}{MIC}\right)}_{50}^{\gamma }}\;$$

where *E*_0_ is the change in log bacterial count after 24 h of incubation compared to the initial inoculum without marbofloxacin (control group). *I_MAX_* is the maximal *log*_10_ reduction in bacterial count after marbofloxacin exposition at 24 h, (*fAUC*_24_/*MIC*)_50_ is the ratio to achieve 50% of *log*_10_ reduction and *γ* is the Hill coefficient, which describes the slope of the curve [36]. 

The inhibitory curves constructed were subsequently modeled and simulated to establish different bactericidal activity profiles defined as PK/PD cutoff. In fact, *fAUC*_24_/*MIC* ratios, which produced 1 *log*_10_ reduction (*E* = −1), 2 *log*_10_ reduction (*E* = −2) and 3 *log*_10_ reduction (*E* = −3), were obtained for each growth medium and inoculum [37]. The values obtained were imported to an excel sheet to be used in subsequent statistical analysis.

### 2.7. Statistical Analysis

The Friedman test was used to compare the differences in *MIC* and *MBC* between growth medium and inoculum sizes. When significant differences were found, a Wilcoxon test was used as a second test (pairwise comparison). Parameters obtained after modeling and simulations of TKC and inhibitory Imax models were analyzed with a generalized mixed-effects model to evaluate differences among groups. 

The link function was selected based on the nature of the data, Akaike information criterion (AIC), and Bayesian information criterion (BIC). Significance was set at 5% throughout (*p* < 0.05). Statistical analysis was performed using Infostat^®^ 2018 (Grupo InfoStat, FCA, Universidad Nacional de Córdoba, Córdoba, Argentina) program. 

## 3. Results

### 3.1. MIC and MBC Determination

*MIC* and *MBC* distribution values for marbofloxacin for each inoculum of *S. aureus* in CAMHB, serum and milk, are shown in Table 1, respectively. Statistical comparison of *MIC* or *MBC* values show significant difference between growth medium and inoculum sizes (*p* < 0.05). These concentrations were higher with increases 2,4-fold in serum and 3,6-fold in milk at high inoculum, respectively (*p* < 0.05).

### 3.2. Time Kill Curves Modeling

TKC modeling with pharmacodynamics population models described the drug concentration–effect relationship through the estimation of different parameters. These models fit well with % RSE values from 3 to 60% for most estimated parameters, as well as a small proportion of outliers close to 5–6% in observations versus predictions and visual predictive check plots, suggesting a good description of the observed data. Parameters obtained after data modeling are described in Table 2, Table 3, respectively. 

From the one population model, maximum bacterial population size *N_MAX_* close to 10^10.1^, 10^9.48^ and 10^11.2^ CFU/mL were obtained with growth rate constants of 0.39, 0.56 and 0.32 1/h for CAMHB, serum and milk, respectively. Only a delayed growth was observed in CAMHB and serum. Maximum killing rates of 0.36, 0.39 and 0.26 1/h with drug concentration to achieve 50% of killing of 0.81, 1.45, and 0.77 mg/L were observed for CAMHB, serum and milk, respectively. 

The inoculum size was the most important covariate, reducing the values of *N_MAX_*, *dk_g_*, *N*_0_, *K_MAX_* and *EC*_50_ at low inoculum, whereas that *EC*_50_ was also influenced by *MIC*, increasing its value with the size of the inoculum. Statistical comparison between growth mediums showed that all parameters were significantly different with P values less than 0.05, with the exception of *N*_0_ and *dk_g_*.

These results suggest that marbofloxacin activity was reduced at high bacterial density, and also with high *MIC* values, especially when *S. aureus* grew in milk.

For the two population bacterial model, maximum bacterial population size close to 10^10.3^, 10^9.57^ and 10^11.1^ CFU/mL were obtained with growth rate constants of 0.40, 0.55 and 0.31 1/h from susceptible, and 0.25, 0.33 and 0.19 1/h for less susceptible populations in CAMHB, serum and milk, respectively. In the same way of the one population model, only a delayed growth was observed in CAMHB and serum. Maximum killing rates of 0.38, 0.39 and 0.24 1/h were observed. Drug concentrations to achieve 50% of killing of 0.73, 1.29 and 0.62 mg/L for susceptible, and 8.85, 6.74 and 8.01 mg/L for less susceptible populations were observed for CAMHB, serum and milk, respectively. 

Statistical comparisons of parameters produced similar results to those observed in the previous one population model. In fact, all parameters were significantly different between growth media with *p* values < 0.05, with the exception of *N*_0_. At low inoculum, parameters of *dk_g_*, *N*_0_, *K_MAX_* and *EC*_50_ were lower compared to high inoculum. On the other hand, *MIC* increased the *EC*_50_ values.

The results from both models indicate that the size of the inoculum, the *MIC* value, as well as the growth medium used, had an important effect on the pharmacodynamics of marbofloxacin against the bacterial population tested. A reduction in activity was observed at high inoculum in all culture mediums.

**Figure 2:**
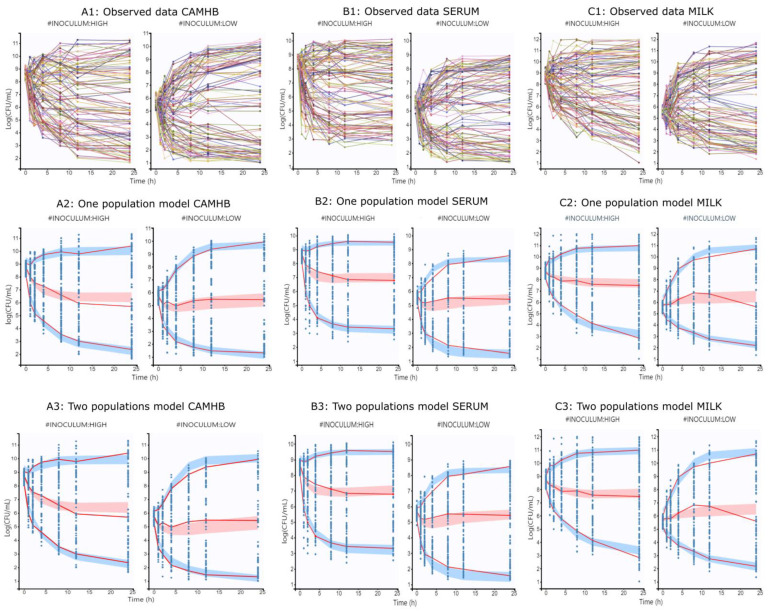
Plots of observed time kill curves stratified by inoculum size. (**A1**): Data from CAHMB; (**A2**): Visual predictive checks plots after modeling CAMHB data with one population model; (**A3**): Visual predictive checks plots after modeling CAMHB data with two populations model; (**B1**): Data from serum; (**B2**): Visual predictive checks plots after modeling serum data with one population model; (**B3**): Visual predictive checks plots after modeling serum data with two population model; (**C1**): Data from milk; (**C2**): Visual predictive checks plots after modeling milk data with one population model; (**C3**): Visual predictive checks plots after modeling milk data with two population model.

The plots of observed data stratified by each inoculum size and growth medium are displayed in Figure 2. Visual predictive checks (VPC) were also included. In the first row of the figure, the observed data of all isolates for each growth medium and inoculum used are shown and named as Figure 2(A1): observed data CAHMB, Figure 2(B1): observed data SERUM and Figure 2(C1): observed data MILK. In the second row, the VPC plots for each medium and inoculum after modeling the data with the one population model are described as Figure 2(A2): one population model CAMHB, Figure 2(B2): one population model SERUM and Figure 2(C2): one population model MILK for CAMHB; serum and milk. Finally, the third row presents the VPC for data analyzed with the two populations bacterial model named M3 and are presented as Figure 2(A3): two population model CAHMB, Figure 2(B3): two population model CAHMB SERUM and Figure 2(C3): two populations model MILK for CAMHB, serum and milk. 

### 3.3. Simulations and PK/PD Relationships

The relationship between PK/PD index (fAUC/*MIC*) and the antibacterial effect (difference of *log*_10_ CFU/mL between 0 and 24 h) were determined using *I_max_* models derived from one and two populations. 

The models obtained presented maximum *log*_10_ reductions of 8.57, 6.75 and 8.88 in CAMHB, serum and milk, respectively, as well as (*fAUC*_24_/*MIC*)_50_ ratios of 172.39, 235.95 and 113.65 from the one population model. Moreover, maximum *log*_10_ reductions of 9.4, 7.39 and 8.72 and (*fAUC*_24_/*MIC*)_50_ ratios of 119, 305.45 and 102.68 were obtained with the two population model. 

The *I_MAX_* models determined for each inoculum and growth medium are shown in Table 4; Table 5, respectively.

**Figure 3:**
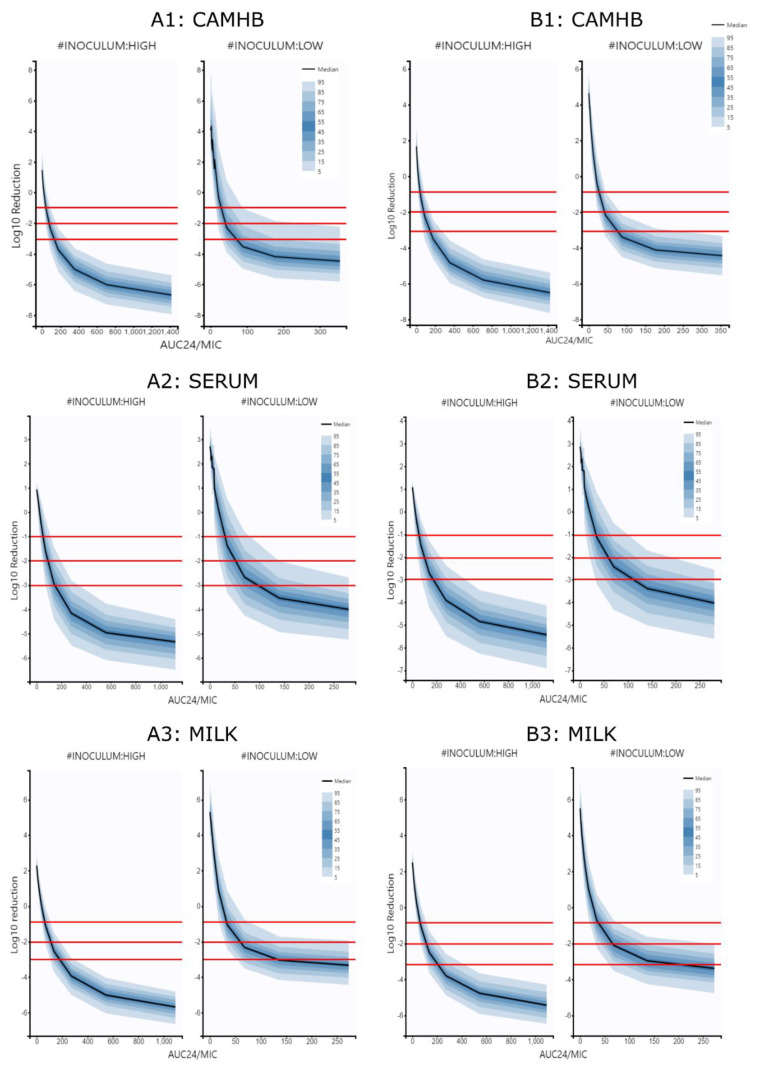
Plots of simulated *log*_10_ reduction data stratified by inoculum size versus *fAUC*_24_/*MIC* values necessary to achieve −3, −2 and −1 *log*_10_ reduction from the initial population. (**A1**): Data from CAMHB after modeling and simulation with one population bacterial model; (**A2**): Data from serum after modeling and simulation with one population bacterial model; (**A3**): data from milk after modeling and simulation with one population bacterial model; (**B1**): Data from CAMHB after modeling and simulation with two population bacterial model; (**B2**): Data from serum after modeling and simulation with two population bacterial model; (**B3**): Data from milk after modeling and simulation with two populations bacterial model. Red horizontal lines represent −1, −2 and −3 *log*_10_ reduction as visual cues.

The *I_MAX_* models were simulated; the curves obtained for each inoculum and growth medium are shown in Figure 3. On the other hand, modeling and simulation of ratios of *fAUC*_24_/*MIC* to achieve *log*_10_ reduction values of −1, −2 and −3 are resented in Table 4, Table 5 and Table 6.

Statistical comparison of the parameters showed that for each inoculum size and growth medium, the PK/PD cutoff points were significantly different (*p* < 0.05). In fact, between mediums, values for milk were higher than serum and CAMHB. Between inoculum sizes, values at high inoculum were different to low inoculum. Finally, the comparisons of PK/PD points between models show that the data obtained using the two populations model were different and higher than the data obtained with the one population model. These results suggest that the inoculum size and the *MIC* values had a great effect on the reduction of bactericidal activity of marbofloxacin.

## 4. Discussion

To the best of our knowledge, this is the first research that has studied the effect of marbofloxacin on a veterinary pathogen, such as *S. aureus* isolated from mastitic goats’ milk using biological fluids as growth medium and two inoculum sizes after non-linear mixed effect modeling analysis.

### 4.1. MIC and MBC Determination

The use of serum and milk increased the *MIC* values determined with respect to artificial media, such as CAMHB. The highest values were obtained in milk, and these findings agree with the observations described with other antimicrobials such as spiramycine, tylosine or kanamycin [21,36]. In fact, some studies have suggested that milk can decrease the antibacterial activity and increase the *MIC* value, probably due to the effect of this fluid on the physicochemical properties of drugs [27,37,38,39]. 

Another important point of this research has been the influence of inoculum size on the activity of marbofloxacin, where an increase of *MIC* values from 2 to 6 has been observed at high bacterial density regarding to low bacterial density (Table 1). In this way, the effect of inoculum of *S. aureus* in artificial mediums and biological fluids as human peritoneal fluid have been studied with pazufloxacin, ciprofloxacin, and levofloxacin [20,31,40,41]. These studies found that the activity of fluoroquinolones was affected with concentrations 2 to 4-fold higher from 10^5^ to 10^8^ CFU/mL unlike with other antimicrobials, such as beta-lactams. These results agree with the data observed in our research, suggesting the existence of a high inoculum effect in the action of fluoroquinolones on bacteria, such as *S. aureus* [18,21,31,40,41].

In this study, blood serum and milk were used as growth mediums and selected to be representative of biological fluids present in animals. However, other mediums as serum milk could have been employed. Previous tests were carried out in our laboratory with milk serum produced in situ, but were discarded due to the difficulty of the procedure, and due to the fact that, to our knowledge, a standardized laboratory protocol to obtain TKC or *MIC* concentrations in milk serum was not found, on the contrary to milk, where multiple references have been read and used [21,36,42].

### 4.2. Drug Effect Modeling

The previously described information has used the *MIC* as a single reference point for the activity of the drug. However, the use of pharmacodynamic models with parameters derived from the Hill function has made it possible to describe the evolution of bacterial killing and the magnitude of the action of marbofloxacin as a function of inoculum size and growth medium. Moreover, data analysis using non-linear mixed effect models has allowed a better understanding of the influence of each parameter and variable to be evaluated. At this point, it is necessary to note that both population models used have shown similar results in some parameters (Table 2; Table 3). For example, *N_MAX_* was higher in milk, intermediate in CAMHB, and lower in serum, but surprisingly, *k_g_* was higher in serum than milk, suggesting that bacteria may grow slower in milk, but reach higher maximum densities due to its better nutritional quality [4]. Moreover, bacterial killing defined as *K_MAX_* was lower in milk, probably due to the reduction in drug activity [37].

The evaluation of more specific parameters of each model showed that *EC*_50_ values in serum were higher than milk, while this was opposite for the two population model for *EC*_50*S*_ and *EC*_50*R*_ values. This is an advantage of the two population model, since it allows to describe the evolution of subpopulations according to their exposure to the antimicrobial [43]. On the other hand, *EC*_50*R*_ values were higher than *EC*_50*S*_ values and *k_gR_* was lower than *k_gS_* values, indicating that the less susceptible population grows more slowly and is less influenced by the action of the drug, as has been widely described in the literature [44,45].

### 4.3. Simulations and PK/PD Cuttoff Points 

Both pharmacodynamic models were used to simulate multiple TKC and the relationship between the effect described as *log*_10_ reduction between 0 to 24 h with the *fAUC*_24_/*MIC* values (Table 4; Table 5). Subsequently, the *I_MAX_* models were used to calculate the corresponding *fAUC*_24_/*MIC* values with different efficacy outputs named −1 *log*_10_ reduction (90% of killing), −2 *log*_10_ reduction (99% of killing) and −3 *log*_10_ reduction (99.9% of killing), displayed in Figure 3 and Table 6.

Statistical comparison of *fAUC*_24_/*MIC* ratios obtained with both models for each growth medium and inoculum tested show significant differences. These findings suggest the PK/PD cutoff points determined are highly dependent on the growth medium and bacterial density [15,23]. The highest values have always been obtained in serum and milk, which are more representative biological fluids than artificial media [13]. For low and high inoculum, median values close to 50–120, 80–160 and 100–175 were obtained in CAMHB, serum, and milk, respectively, and fall in the range of standardized values for fluoroquinolones of 50–125 that have been widely described [9]. However, it is necessary to indicate that the numerical values recommended to establish the PK/PD cutoff points for concentration-dependent antimicrobial drugs as fluoroquinolones have been generated in experimental infections in laboratory animals or in human clinical trials [23,44]; thus, these observed ratios might not be applicable to goat infections or to other infections in ruminants [45,46,47,48,49]. In the same way, *fAUC*/*MIC* ratios for ruminant species have been described in different experiments with TKC with values lower than 100 or higher than 180, especially with high bacterial densities [15,23]. In addition, other factors, such as the immunocompetence of the host and the post-antibiotic effect of fluoroquinolones, should be considered cautiously with these ratios [47]. 

Determination of effective PK/PD ratios with static in vitro models is a useful tool; however, it has known limitations. In fact, static assays do not take into account the inmune and granulocyte response of the host, and only the antimicrobial bacterial killing effect is evaluated. However, in vivo infection models combining antimicrobials and granulocyte killing have reported a saturable effect with maximal killing rate close to −1 and −2 *log*_10_ CFU/g reduction [50,51]. These studies have shown that an early initial reduction of at least −2 *log*_10_ (99% of killing) was neccesary to avoid granulocyte saturation, and this pharmacodynamic outcome could be adequate for immunocompetent animals [52]. On the other hand, the contribution of post antibiotic effect (PAE) on bacterial killling should be considered [10]. Some studies have shown PAE values in *S. aureus* and other bacteria exposed to fluoroquinolones ranging between 5 and 22 h after exposure to the antimicrobial [10,53,54]. For that reason, in vitro *fAUC*_24_/*MIC* ratios obtained in this study should be considered as starting points for subsequent clinical studies where the values achieved could be lower given the effect of the immune system and the PAE of fluoroquinolones [10,52,53,54].

## 5. Conclusions

In this study, the bactericidal activity of marbofloxacin against *S. aureus* isolates was determined using different approaches, such as *MIC* and PK/PD modeling. The main findings of this research suggest that firstly, the combined use of biological fluids such as serum or milk with different inoculum sizes showed that the ratios to predict clinical efficacy with fluoroquinolones are highly dependent on the growth medium and bacterial density. Secondly, the determination of *MIC* in biological fluids may be more representative than in artificial media, since they have different rates of bacterial growth and high influence of protein binding. Thirdly, the use of population pharmacodynamic models made it possible to describe the evolution of the drug–bacteria interaction as a function of covariates, such as growth medium, *MIC* or bacterial density, and subsequently allowed multiple scenarios to be simulated to evaluate cutoff points, and, finally, the determined *fAUC*_24_/*MIC* ratios indicate that for each antimicrobial and pathogen, the use of optimal growth media such as serum or milk and variable bacterial densities produced higher ratios than in artificial media such as CAMHB.

However, this research has been carried out with static in vitro models where the variations in the concentration of the antimicrobial have not been taken into account, mainly due to the limitation of the use of biological fluids in continuous experiments. Moreover, this research highlights the importance of selecting an optimal growth medium and inoculum size to evaluate the bacterial drug interaction; further investigations with other drugs and bacteria are necessary in this context. 

## Figures and Tables

**Table 1:** *MIC* and *MBC* distribution values for marbofloxacin against *S. aureus* isolated from mastitic goats´ milk (n = 12). Values presented for each growth medium tested for low (≈10^5^) and high (≈10^8^) inoculum, respectively.

MEDIUM	CAMHB				Serum				Milk			
INOCULUM	LOW		HIGH		LOW		HIGH		LOW		HIGH	
Drug (mg/L)	*MIC*	*MBC*	*MIC*	*MBC*	*MIC*	*MBC*	*MIC*	*MBC*	*MIC*	*MBC*	*MIC*	*MBC*
0.125	5		2									
0.25	4	1			1		2					
0.5	3	6	7	2	6		5		5		3	
1		2	3	5	5	3	5	4	7	1	9	
2		3		3		3		5		3		2
4				2		6		3		8		8
8												2

*MIC* = minimum inhibitory concentration, mg/L; *MBC* = maximum inhibitory concentration. According to 2017 CLSI guidelines [19].

**Table 2:** Final parameters from one population bacterial model for each growth medium used. Data presented as estimates with % RSE, including inter-individual variability (IIV).

	CAMHB		Serum		Milk	
Parameters	Estimates	IIV	Estimates	IIV	Estimates	IIV
*N_MAX_* (*log* CFU/mL)	10.1 (0.85)	0.04 (17.1)	9.48 (0.62)	0.01 (64.4)	11.2 (0.97)	0.033 (21.2)
*k_g_* (1/h)	0.39 (3.13)	0.06 (57.0)	0.56 (2.96)	0.06 (50.6)	0.32 (3.26)	0.07 (51.4)
*dk_g_* (1/h)	2.41 (17.3)	0.79 (8.47)	2.37 (17.6)	1.26 (7.01)		
*N*_0_ (*log* CFU/mL)	8.66 (0.50)	0.014 (81.8)	8.58 (0.48)	0.02 (31.2)	8.7 (0.5)	0.02 (39.0)
*γ*	0.92 (2.69)	0.13 (16.2)	1.23 (2.72)	0.18 (14.7)	1.13 (2.86)	0.15 (16.8)
*K_MAX_* (1/h)	0.36 (3.1)	0.04 (110)	0.39 (3.59)	0.06(49.8)	0.26 (3.04)	0.05 (82.2)
*EC*_50_ (mg/L)	0.81 (12.8)	0.29 (14.7)	1.45 (12.0)	0.25 (15.4)	0.77 (14.9)	0.26 (17.5)
Covariate estimates						
β_ *N_MAX_*__INOCULUM_LOW_			−0.13 (7.26)			
β_*dk_g_*__INOCULUM_LOW_	−1.11 (17.5)		−0.75 (30.2)			
β_*N_0_*__INOCULUM_LOW_	−0.424 (1.96)		−0.42 (1.89)		−0.42 (2.07)	
β_*γ*__INOCULUM_LOW_	0.315 (15.2)					
β_*K_MAX_*__INOCULUM_LOW_			0.20 (16.5)			
β_*EC*_50___INOCULUM_LOW_	−1.89 (5.28)		−1.70 (5.97)		−1.82 (5.19)	
β_*EC*_50__*_MIC_*	2.25 (6.88)		1.07 (11.3)		1.74 (8.32)	

*N_MAX_*: maximum total bacterial population. *k_g_*: bacterial growth rate constant. *dk_g_*: delayed growth rate constant observed in the initial phases. *N*_0_: initial bacterial population. *γ*: sigmodicity of the concentration effect curve. *K_MAX_*: drug killing rate constant. *EC*_50_: drug concentration to achieve 50% of killing. *β*_ *N_MAX_*__INOCULUM_LOW_*:* covariate parameter to effect of inoculum on *N_MAX_*. *β*_ *dk_g_*__INOCULUM_LOW_*:* covariate parameter to effect of inoculum on *dk_g_*. *β*_ *N_0_*__INOCULUM_LOW_*:* covariate parameter to effect of inoculum on N_0_. *β*_*γ* __INOCULUM_LOW_*:* covariate parameter to effect of inoculum on γ. *β*_ *K_MAX_*__INOCULUM_LOW_*:* covariate parameter to effect of inoculum on *K_MAX_*. β_*EC*_50___INOCULUM_LOW_ covariate parameter to effect of inoculum on EC_50_. *β*_ *EC*_50__*_MIC_:* covariate parameter to effect of *MIC* on *EC*_50_.

**Table 3:** Final parameters from two population bacterial model for each growth medium used. Data presented as estimates with % RSE, including inter-individual variability (IIV).

	CAMHB		Serum		Milk	
Parameters	Estimates	IIV	Estimates	IIV	Estimates	IIV
*N_MAX_* (*log* CFU/mL)	10.3 (1.01)	0.05 (15.9)	9.57 (0.69)	0.02 (48.1)	11.1 (0.92)	0.04 (19.1)
*k_gS_* (1/h)	0.40 (3.33)	0.04 (138.0)	0.55 (3.11)	0.09 (28.2)	0.31 (3.28)	0.08 (50.6)
*f_g_*	0.62 (0.95)	1.07 (82.3)	0.60 (0.32)	1.67 (106)	0.61 (3.29)	1.99 (72)
*dk_g_* (1/h)	2.52 (18.0)	0.885 (8.77)	1.98 (18.6)	1.11 (7.95)		
*N*_0_ (*log* CFU/mL)	6.84 (0.32)	0.01 (65.1)	6.78 (0.30)	0.01 (24.7)	6.84 (0.32)	0.01 (27.8)
*γ*	0.79 (4.60)	0.13 (45.9)	1.07 (3.20)	0.18 (14.3)	1.06 (5.74)	0.15 (46.9)
*K_MAX_* (1/h)	0.38 (3.63)	0.04 (117)	0.39 (4.16)	0.06 (59.3)	0.24 (4.19)	0.05 (95.1)
*EC*_50*S*_ (mg/L)	0.73 (14.6)	0.315 (15)	1.29 (13.6)	0.22 (24)	0.62 (15.7)	0.1 (28.4)
*f_s_*	4.02 (0.74)	2.28 (150)	3.51 (0.7)	2.67 (155)	4.6 (5.59)	1.54 (66.3)
Secondary parameters						
*EC*_50*R*_	8.85 (8.43)		6.74 (3.97)		8.01 (4.96)	
*kg_R_*	0.25 (0.08)		0.33 (0.38)		0.19 (0.20)	
Covariate estimates						
β_ *N_MAX_*__INOCULUM_LOW_			−0.127(8.3)			
β_*dk_g_*__INOCULUM_LOW_	−1.02 (20.6)		−0.62 (37)			
β_*N*_0___INOCULUM_LOW_	−0.246 (1.97)		−0.243 (1.9)		−0.25 (1.98)	
β_*γ*__INOCULUM_LOW_	0.417 (14.9)					
β_*K_MAX_*__INOCULUM_LOW_	−1.94 (5.47)		0.23 (18.1)		0.17 (20.3)	
β_*EC*_50___INOCULUM_LOW_	2.24 (7.33)		−1.54 (8.21)		−1.46 (7.61)	
β_*EC*_50__*_MIC_*			1.08 (11.7)		1.71 (7.96)	

N_MAX_: maximum total bacterial population. *k_gS_*: bacterial growth rate constant for susceptible population. *f_g_*: proportional factor between two populations rate constants. *dk_g_*:, delayed growth rate constant observed in the initial phases. *N*_0_: initial bacterial population. *γ*: sigmodicity of the concentration effect curve. *K_MAX_*: drug killing rate constant. *EC*_50*S*_: drug concentration to achieve 50% of killing of susceptible population. *f_S_*: proportional factor between two *EC*_50_ values. *EC*_50*R*_: drug concentration to achieve 50% of killing of less susceptible population. *k_gR_*: bacterial growth rate constant for less susceptible population. *β*_ *N_MAX_*__INOCULUM_LOW_*:* covariate parameter to effect of inoculum on *N_MAX_*. *β*_ *dk_g_*__INOCULUM_LOW_*:* covariate parameter to effect of inoculum on *dk_g_*. *β*_*N*_0___INOCULUM_LOW_*:* covariate parameter to effect of inoculum on *N*_0_. *β*_*γ*__INOCULUM_LOW_*:* covariate parameter to effect of inoculum on *γ*. *β*_ *K_MAX_*__INOCULUM_LOW_*:* covariate parameter to effect of inoculum on K_MAX_. β_*EC*_50___INOCULUM_LOW_ covariate parameter to effect of inoculum on *EC*_50_. *β*_ *EC*_50__*_MIC_:* covariate parameter to effect of *MIC* on *EC*_50_.

**Table 4:** Final parameters from inhibitory I_MAX_ model obtained after time kill curves simulated with one population bacterial model for each growth medium used. Data presented as estimates with % RSE, including inter-individual variability (IIV).

	CAMHB		Serum		Milk	
Parameters	Estimates	IIV	Estimates	IIV	Estimates	IIV
*E*_0_	0.92 (4.16)	0.33 (4.10)	0.94 (1.51)	0.15 (4.51)	2.29 (1.38)	0.16 (4.52)
*I_MAX_*	8.57 (1.06)	0.11 (4.13)	6.75 (0.92)	0.09 (4.61)	8.88 (0.50)	0.07 (4.70)
*γ*	0.88 (0.75)	0.10 (4.24)	1.23 (1.31)	0.21 (4.51)	0.97 (0.73)	0.08 (4.70)
*EC*_50_ (mg/L)	172.39 (6.03)	0.38 (4.10)	235.95 (6.24)	0.35 (4.50)	113.65 (2.76)	0.27 (4.51)
Covariate estimates						
β_*E*_0___INOCULUM_LOW_	1.29 (2.07)		1.05 (1.85)		0.83 (1.90)	
β_*E*_0__*_MIC_*	0.79 (8.60)					
β_*γ*__INOCULUM_LOW_	0.46 (2.15)				0.31(2.99)	
β_*I_max_*_*_MIC_*	0.12 (15.8)					
β_*EC*_50___INOCULUM_LOW_	−1.93 (2.63)		−1.59 (2.96)		−1.91 (1.86)	
β_*EC*_50__*_MIC_*	−0.55 (16.3)		−1.05 (6.59)			

E_0_: change in *log*_10_ bacterial count after 24 h of incubation compared to the initial inoculum without marbofloxacin (control group). *I_MAX_*: maximal *log*_10_ reduction in bacterial count after marbofloxacin exposition at 24 h. *γ*: sigmodicity of the concentration effect curve. *EC*_50_: free *AUC*_24_/*MIC* ratio for each concentration tested expressed as *AUC*_24_ values. *β*_ *E*_0___INOCULUM_LOW_*:* covariate parameter to effect of inoculum on *E*_0_. *β*_*E*_0__*_MIC_:* covariate parameter to effect of *MIC* on N_0_. *β*_*γ*__INOCULUM_LOW_*:* covariate parameter to effect of inoculum on γ. *β*_ *I_MAX_*_*_MIC_:* covariate parameter to effect of *MIC* on E_MAX_. β_*EC*_50___INOCULUM_LOW_ covariate parameter to effect of inoculum on *EC*_50_. *β*_ *EC*_50__*_MIC_:* covariate parameter to effect of *MIC* on *EC*_50_.

**Table 5:** Final parameters from inhibitory I_MAX_ model obtained after time kill curves simulated with two population bacterial model for each growth medium used. Data presented as estimates with % RSE, including inter-individual variability (IIV).

	CAMHB		Serum		Milk	
Parameters	Estimates	IIV	Estimates	IIV	Estimates	IIV
*E*_0_	1.76 (3.31)	0.02 (98.1)	1.08 (1.53)	0.15 (4.61)	2.51 (1.46)	0.08 (4.66)
*I_max_*	9.4 (0.79)	0.09 (155)	7.39 (1.06)	0.10 (4.97)	8.72 (0.76)	0.07 (4.66)
*γ*	0.81 (1.58)	0.04 (69.2)	1.01 (1.44)	0.14 (4.79)	0.97 (1.33)	0.13 (4.59)
*EC*_50_ (mg/L)	119 (5.93)	0.20 (6.88)	305.45 (6.64)	0.37 (4.58)	102.68 (2.42)	0.24 (4.53)
Covariate estimates						
β_*E*_0___INOCULUM_LOW_	1.0 (2.75)		0.97 (2.04)		0.78 (2.41)	
β_*E*_0__*_MIC_*	−0.112 (33)					
β_*γ*__INOCULUM_LOW_	0.40 (4.27)					
β_*EC*_50___INOCULUM_LOW_	−1.88 (2.65)		−1.68 (2.98)		−1.74 (1.79)	
β_*EC*_50__*_MIC_*	0.22 (35.2)		−1.07 (6.83)			

E_0_: change in *log*_10_ bacterial count after 24 h of incubation compared to the initial inoculum without marbofloxacin (control group). *I_MAX_*: maximal *log*_10_ reduction in bacterial count after marbofloxacin exposition at 24 h. *γ*: sigmodicity of the concentration effect curve. *EC*_50_: free *AUC*_24_/*MIC* ratio for each concentration tested expressed as *AUC*_24_ values. *β*_ *E*_0___INOCULUM_LOW_*:* covariate parameter to effect of inoculum on E_0_. *β*_*E*_0__*_MIC_:* covariate parameter to effect of *MIC* on N_0_. *β*_*γ*__INOCULUM_LOW_*:* covariate parameter to effect of inoculum on γ. β_*EC*_50___INOCULUM_LOW_ covariate parameter to effect of inoculum on *EC*_50_. *β*_ *EC*_50__*_MIC_:* covariate parameter to effect of *MIC* on *EC*_50_.

**Table 6:** PK/PD breakpoint expressed as *fAUC*_24_/*MIC* necessary to achieve a *log*_10_ reduction of −1, −2 and −3 from the initial populations. Data are presented as median with 5th and 95th percentiles from one and two population bacterial models, respectively.

			One Population Model	Two Populations Model
Medium	Inoculum	*log*_10_ Reduction	*fAUC*_24_/*MIC*	*fAUC*_24_/*MIC*
CAMHB	HIGH	−1	42.68 (21.57–87.97)	43.15 (29.57–58.53)
CAMHB	HIGH	−2	74.49 (39.34–150.4)	78.04 (54.17–106.54)
CAMHB	HIGH	−3	123.96 (64.69–242.95)	133.53 (92.98–184.09)
CAMHB	LOW	−1	27.76 (13.19–85.61)	27.17 (20.08–38.9)
CAMHB	LOW	−2	39.42 (18.33–127.66)	40.62 (29.92–58.91)
CAMHB	LOW	−3	59.84 (27.39–194.15)	66.65 (48.04–97.65)
SERUM	HIGH	−1	50.25 (24.9–109.51)	52.65 (23.76–114.93)
SERUM	HIGH	−2	85.52 (44.91–187.63)	97.31 (43.89–222.42)
SERUM	HIGH	−3	144.65 (71.15–316.14)	166.2 (73.71–409.32)
SERUM	LOW	−1	29.43 (11.95–76.64)	33.03 (12.57–91.57)
SERUM	LOW	−2	47.3 (18.68–139.08)	54.38 (19.7–172.83)
SERUM	LOW	−3	86.77 (30.22–377.42)	101.91 (31.7–449.27)
MILK	HIGH	−1	66.27 (40.93–107.98)	67.67 (41.49–116.83)
MILK	HIGH	−2	105.47 (65.26–172.06)	108.01 (66.71–193.43)
MILK	HIGH	−3	168.7 (100.16–282.9)	175.58 (103.17–344.51)
MILK	LOW	−1	33.21 (18.38–76.76)	38.46 (20.41–91.36)
MILK	LOW	−2	54.68 (27.69–160.3)	63.81 (31.06–198.05)
MILK	LOW	−3	106.65 (45.01–527.62)	123.31 (48.98–971.07)

## Data Availability

The data presented in this study are available on request from the corresponding author.

## Supplementary Materials

The following are available online at https://www.mdpi.com/article/10.3390/antibiotics10111290/s1, Figure S1: TKC from 0 × MIC to 16 × MIC are shown with 7 sampling points producing 63 data points in total title, Figure S2: TKC from 12 *S.auerus* isolates were observed from 0 × MIC to 16 × MIC. In this profile, corresponding to milk at low inoculum, 12 isolates with 9 TKC per isolate and 7 data points produced a data set with 756 points, Table S1: Isolates used in this study.
