# Supplementary material for: Effects of Growth Medium and Inoculum Size on Pharmacodynamics Activity of Marbofloxacin against Staphylococcus aureus Isolated from Caprine Clinical Mastitis
Source: Antibiotics (Basel). 2021 Oct 22;10(11):1290. doi: 10.3390/antibiotics10111290 (PMC8614650; doi:10.3390/antibiotics10111290)
Supplement: Supplementary file 1 [file antibiotics-10-01290-s001.zip › antibiotics-1338577-supplementary.pdf]

antibiotics

MDPI

*Supplementary material.*

**Isolated**

Twelve *S. aureus* isolated from milk of goats with clinical mastitis from different animals and farms in Spain were *generously supplied by* Exopol laboratory S.L. and are shown in Table S1. Isolated by incubation into agar plates and identified by PCR methods.

**Table S1.** Isolates used in this study.

| Isolates | Isolation date | Spanish Region | | Isolation date | Spanish Region |
|---|---|---|---|---|---|
| Isolate 1 | 17/08/2012 | Toledo | Isolate 7 | 17/04/2012 | Fuerteventura |
| Isolate 2 | 26/07/2012 | Sevilla | Isolate 8 | 17/04/2012 | Fuerteventura |
| Isolate 3 | 11/07/2012 | Castelo Branco[1] | Isolate 9 | 23/03/2012 | La Coruña |
| Isolate 4 | 07/06/2012 | Toledo | Isolate 10 | 13/03/2012 | Cáceres |
| Isolate 5 | 06/06/2012 | Fuerteventura | Isolate 11 | 27/02/2012 | Cáceres |
| Isolate 6 | 31/05/2012 | Toledo | Isolate 12 | 27/01/2012 | Cáceres |

[1] Strain obtained in Castelo Branco (Portugal).

**Time kill curves description.**

For each *S.aureus* isolate different time kill curves (TKC) were determined al low and high inoculum in CAMHB, blood serum and milk. The methodology of TKC determination performed in our laboratory is described below using isolate 9 in milk at low inoculum as example.

After MIC determination, a single isolate was used for each TKC, and from this, different data time points were sampled, as indicated here:
TKC from Isolate 9 at 0xMIC: sampling time: 0, 1, 2, 4, 8, 12 and 24 h
TKC from Isolate 9 at 0.5xMIC: sampling time: 0, 1, 2, 4, 8, 12 and 24 h
TKC from Isolate 9: at 1xMIC sampling time: 0, 1, 2, 4, 8, 12 and 24 h
TKC from Isolate 9: at 2xMIC sampling time: 0, 1, 2, 4, 8, 12 and 24 h
TKC from Isolate 9: at 4xMIC sampling time: 0, 1, 2, 4, 8, 12 and 24 h
TKC from Isolate 9: at 8xMIC sampling time: 0, 1, 2, 4, 8, 12 and 24 h
TKC from Isolate 9: at 16xMIC sampling time: 0, 1, 2, 4, 8, 12 and 24 h
TKC from Isolate 9: at 32xMIC sampling time: 0, 1, 2, 4, 8, 12 and 24 h
TKC from Isolate 9: at 64xMIC sampling time: 0, 1, 2, 4, 8, 12 and 24 h

In conclusion, isolate 9 produces 9 TKC with 7 data points per curve (63 data points in total).

This experience was repeated on another different day, so that, the geometric mean was calculated to obtain the final average data for each time point. Finally, the data set of isolate 9 was obtained and is graphically presented below:

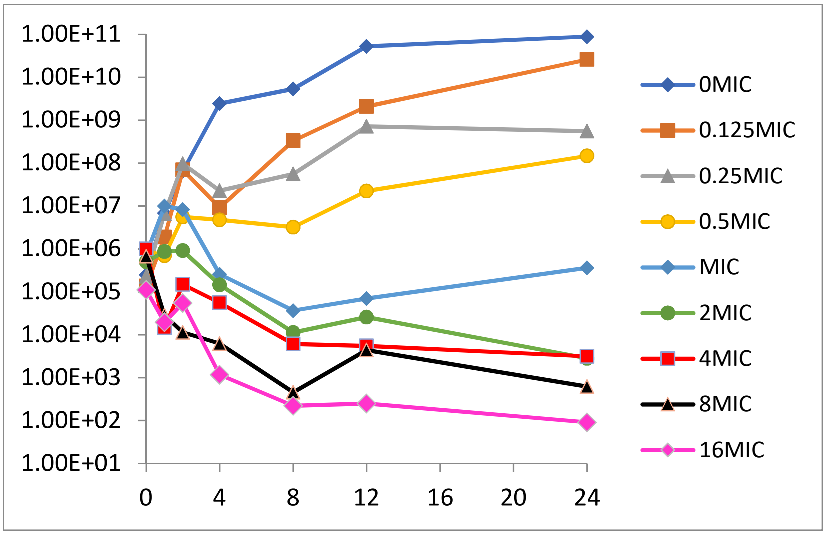


**Figure S1.** TKC from 0 x MIC to 16 x MIC are shown with 7 sampling points producing 63 data points in total title.

In this Figure S1 TKC from 0xMIC to 16xMIC are shown with 7 sampling points producing 63 data points in total.

However, all the isolates (with its respective TKC profile) were represented graphically together, since individually there are too many curves as indicated in the next figure (see below).

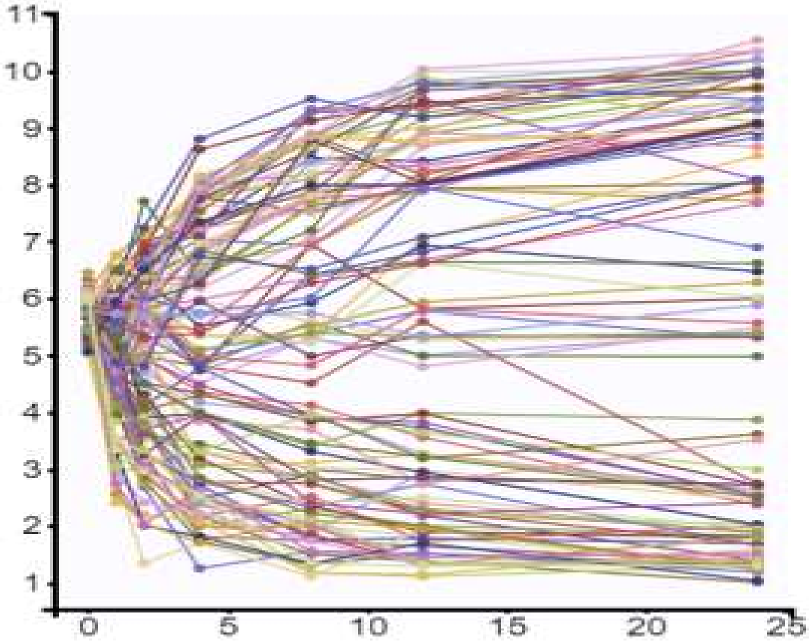


**Figure S2.** TKC from 12 *S. auerus* isolates were observed from 0 x MIC to 16 x MIC. In this profile, corresponding to milk at low inoculum, 12 isolates with 9 TKC per isolate and 7 data points produced a data set with 756 points.

In this figure a lot of TKC from 12 *S. auerus* isolates were observed from 0xMIC to 16xMIC. In this profile, corresponding to milk at low inoculum, 12 isolates with 9 TKC per isolate and 7 data points produced a data set with 756 points.

In this research, three growth medium and two inoculum sizes were used. Finally six data sets were obtained as

Data set 1 CAMHB Low Inoculum:
12 isolates with 9 TKC and 7 data points per TKC = 756 points
Data set 2 CAMHB High Inoculum:
12 isolates with 9 TKC and 7 data points per TKC = 756 points
Data set 3 Serum Low Inoculum:
12 isolates with 9 TKC and 7 data points per TKC = 756 points
Data set 4 Serum High Inoculum:
12 isolates with 9 TKC and 7 data points per TKC = 756 points
Data set 5 Milk Low Inoculum:
12 isolates with 9 TKC and 7 data points per TKC = 756 points
Data set 6 Milk High Inoculum:
12 isolates with 9 TKC and 7 data points per TKC = 756 points

These datasets were organized and tabulated to be analyzed with the population models.

**Population modeling and data tabulation.**

After TKC determination, all data sets obtained were organized and classified in the three groups:

Group 1:

TKC from CAMHB at low and high inoculum, resulting in 1512 points to analyze.

Group 2:

TKC from serum at low and high inoculum, resulting in 1512 points to analyze.

Group 3:

TKC from milk at low and high inoculum, resulting in 1512 points to analyze.

From each group, MIC was used as continuous covariate, and inoculum size (low or high) as categorical covariate.

For example, data from group 1 were modeled with one population model in a first phase, following a second analysis with two populations model. Data from group 2 and 3 were analyzed in the same way.

After modeling the data, different figures were obtained and included into a multiple figure named figure 2 into the manuscript. Parameters obtained after modeling are shown in tables 2 and 3.
